# Transportation barriers to care among frequent health care users during the COVID pandemic

**DOI:** 10.1186/s12889-022-14149-x

**Published:** 2022-09-20

**Authors:** Abigail L. Cochran, Noreen C. McDonald, Lauren Prunkl, Emma Vinella-Brusher, Jueyu Wang, Lindsay Oluyede, Mary Wolfe

**Affiliations:** 1 Department of City and Regional Planning, University of North Carolina at Chapel Hill, New East Building, CB# 3140, 223 E Cameron Ave, NC 27599 Chapel Hill, USA; 2 Community and Regional Planning Program, College of Architecture, University of Nebraska–Lincoln, 217 Architecture Hall, NE 68588 Lincoln, USA; 3 Kittelson & Associates, Inc., 212 S Tryon St Suite 1650, Charlotte, NC 28281 USA; 4 Texas A&M Transportation Institute, Texas A&M University System, 505 E Huntland Dr, Austin, TX 78752 USA; 5 School of Geographical Sciences and Urban Planning, Arizona State University, Lattie F. Coor Hall, 975 S Myrtle Ave, Tempe, AZ 85281 USA; 6 Center for Health Equity Research, University of North Carolina at Chapel Hill, 323 MacNider Hall, 333 South Columbia Street, NC 27599 Chapel Hill, USA

**Keywords:** COVID-19, Health care transportation, Health equity, Health services accessibility, Medicaid, Medicare, Non-emergency medical transportation, Social determinants of health, Survey study, Transportation barriers

## Abstract

**Background:**

Transportation problems are known barriers to health care and can result in late arrivals and delayed or missed care. Groups already prone to greater social and economic disadvantage, including low-income individuals and people with chronic conditions, encounter more transportation barriers and experience greater negative health care consequences. Addressing transportation barriers is important not only for mitigating adverse health care outcomes among patients, but also for avoiding additional costs to the health care system. In this study, we investigate transportation barriers to accessing health care services during the COVID-19 pandemic among high-frequency health care users.

**Methods:**

A web-based survey was administered to North Carolina residents aged 18 and older in the UNC Health system who were enrolled in Medicaid or Medicare and had at least six outpatient medical appointments in the past year. 323 complete responses were analyzed to investigate the prevalence of reporting transportation barriers that resulted in having arrived late to, delayed, or missed care, as well as relationships between demographic and other independent variables and transportation barriers. Qualitative analyses were performed on text response data to explain transportation barriers.

**Results:**

Approximately 1 in 3 respondents experienced transportation barriers to health care between June 2020 and June 2021. Multivariate logistic regressions indicate individuals aged 18–64, people with disabilities, and people without a household vehicle were significantly more likely to encounter transportation barriers. Costs of traveling for medical appointments and a lack of driver or car availability emerged as major transportation barriers; however, respondents explained that barriers were often complex, involving circumstantial problems related to one’s ability to access and pay for transportation as well as to personal health.

**Conclusions:**

To address transportation barriers, we recommend more coordination between transportation and health professionals and the implementation of programs that expand access to and improve patient awareness of health care mobility services. We also recommend transportation and health entities direct resources to address transportation barriers equitably, as barriers disproportionately burden younger adults under age 65 enrolled in public insurance programs.

## Background

Transportation barriers create obstacles to health care and are known to result in delayed and missed appointments as well as medication use [1]. 5.8 million people in the United States delayed medical care in 2017 because they did not have transportation [2]. Groups that are already prone to greater social and economic disadvantage, including individuals who are poor and/or under or uninsured and who have chronic conditions, are more likely to encounter transportation barriers to care and experience negative health consequences [2–5]. Addressing transportation barriers that result in delayed or missed care is important not only for mitigating adverse health care outcomes among patients, but also for avoiding additional costs to the health care system stemming from increased use of emergency departments and hospitalizations [6–9].

The COVID-19 pandemic widely disrupted health and transportation systems in the US. Beginning in March 2020, many health systems deferred non-emergency medical procedures and other elective care [10]. The postponement of medical care remained high throughout 2020. Giannouchos et al. found that 26.9% of adults 18–64 reported having foregone medical care from August to December 2020, while 35.9% reported having delayed care [11]. Though in-person appointments have resumed, many fields face unprecedented patient care backlogs [12]. Public transportation systems reduced service in many cases during the early months of the pandemic response, and riders reported hesitation using public or shared modes due to concerns about infection risk [13]. This likely exacerbated transportation barriers to health care for people without access to a personal vehicle, including some individuals with disabilities [14].

Using mobile device data to explore temporal patterns in visits to health care points of interest during 2020, Wang et al. found census block groups in North Carolina with higher population density and those with higher percentages of older adults, low-income individuals, racial and ethnic minorities, and people without household vehicles had lower rates of medical visits during the pandemic and experienced a slower recovery in visits after the state’s most restrictive lockdown period spanning from mid-March to May 2020 [15]. This may indicate that problems accessing transportation and other barriers to health care are disproportionately affecting populations already known to experience transportation and health disadvantages, particularly during the pandemic.

Synthesizing knowledge on transportation access to health care during the pandemic, Chen et al. found that some patients seeking care required additional support, particularly those who already experienced socioeconomic and transportation disadvantages such as low-income individuals, people of color, and people with disabilities [10]. They were not always able to rely on others or on public transportation for rides like they had in the past, experienced added challenges because of economic hardship due to COVID-19, and found it more difficult to fulfill their health care needs using telemedicine. The authors suggested that partnerships between health and transportation systems hold promise for addressing transportation barriers during and after the pandemic but noted that these partnerships, i.e., arrangements to provide non-emergency medical transportation (NEMT) services, are largely limited to low-income patients enrolled in Medicaid. They reviewed alternative strategies for addressing patients’ transportation needs, including new models for providing NEMT though health care partnerships with ridehailing companies (e.g., Uber and Lyft) as well as innovations in health care coordination and policy, and concluded that such strategies might reduce transportation barriers and promote equity in health care access.

In this study, drawing on results of a survey conducted with high-frequency health care users in North Carolina, we investigate transportation barriers to accessing health care during the COVID-19 pandemic. We examine if and how adult North Carolina residents in the UNC Health Care (“UNC Health”) system who had at least six outpatient medical appointments between April 2020 and April 2021 and are enrolled in Medicaid or Medicare encountered transportation barriers. We explain how barriers affected respondents’ care due to having delayed, missed, or arrived more than 20 minutes late to appointments because of transportation problems. Using demographic and other information collected for respondents, we analyze what factors were associated with reporting transportation barriers that resulted in negative care outcomes. We conclude by making recommendations regarding strategies to address transportation barriers that might meet the needs of high-frequency health care users who have greater health care-related transportation burdens and are more likely to encounter transportation barriers to care.

## Methods

### Sampling and recruitment

The goal of this research was to examine transportation-related barriers to accessing health care among groups known to have greater health care and health care-related transportation burdens, including low-income people, older adults, and individuals with chronic conditions. We thus purposively sampled from these groups, i.e., people with low incomes and those aged 65 and older, and individuals that needed to access care multiple times during the previous year. We recruited participants using data provided by the Carolina Data Warehouse for Health (CDW-H), a central data repository containing clinical, research, and administrative data sourced from the UNC Health system. UNC Health is a not-for-profit medical system owned by the state of North Carolina; while based in Chapel Hill, UNC Health operates hospitals and medical practices across the state. At the recruitment stage we selected from 34,387 individuals to generate a sample of ~ 15,000 people who met the following inclusion criteria: (1) have Medicaid or Medicare as their primary insurance; (2) are North Carolina residents; (3) are over age 18; (4) have a valid email address; and (5) had six or more outpatient visits between April 2020 and April 2021.

Our first inclusion criterion, having Medicaid or Medicare as one’s primary insurance, predictably skewed our sample toward people aged 65 and older. To achieve greater representation of adults aged 18–64, we oversampled from this age group. We then quota-sampled amongst older adults so that the recruitment sample of individuals aged 65–79 and over 80 approximately matched the population of North Carolina; 15.9% of the state population is aged 65–79 and 4.5% is 80 plus according to recent Census estimates [16]. A total of 14,723 people were ultimately included in the recruitment sample, comprising 6945 individuals aged 18–64; 6201 individuals aged 65–79; and 1577 individuals aged 80 or older (Table 1, column 1).

**Table 1:** Summary statistics for recruitment and study samples

	Recruitment sample *N = 14,723*	Study sample *N = 323*
**Age**		
18–64	6945 (47.2%)	125 (38.7%)
65–79	6201 (42.1%)	171 (52.9%)
80 plus	1577 (10.7%)	27 (8.4%)
**Gender**		
Female	9199 (62.5%)	187 (57.9%)
Male	5524 (37.5%)	136 (42.1%)
**Race or Ethnicity**		
White or Caucasian	10,212 (69.4%)	267 (82.7%)
Black or African American	3716 (25.2%)	42 (13.0%)
Asian	129 (0.9%)	1 (0.3%)
American Indian or Alaska Native	67 (0.5%)	2 (0.6%)
Native Hawaiian or Other Pacific Islander	6 (0.0%)	–
Hispanic, Latino, or Spanish	–	6 (1.9%)
Other Race or Multiracial/Multiethnic	489 (3.3%)	5 (1.5%)
Unknown	79 (0.5%)	–
Declined to Answer	25 (0.2%)	–

### Data collection

The research study protocol, including all data collection instruments, was reviewed and approved by the Institutional Review Board at the University of North Carolina at Chapel Hill. Data were collected using REDCap, a secure web platform for managing online databases and surveys. We sent an email invitation to participate in our web-based survey and up to three reminders. The recruitment emails announced that respondents would be entered into a drawing to receive one of twenty $50 gift cards. Respondents completed an eligibility screener to confirm they met the inclusion criteria, a consent form, and an optional HIPAA authorization. Survey data collection occurred between June 21 and July 23, 2021. Upon completion of data collection, 728 individuals completed the eligibility screener and 433 completed the consent form. 383 individuals at least partially completed the survey questionnaire, representing a 2.6% response rate.

### Study sample

323 eligible respondents who answered all questions analyzed in this study were included in the study sample (Table 1, column 2). Like the recruitment sample, the study sample included greater representation of adults aged 65–79 (52.9%) than other age groups; 38.7% of respondents were aged 18–64 and 8.4% were aged 80 years or older. Similarly, as with the recruitment sample, a majority of respondents (57.9%) identified their gender as female. A greater percentage of individuals in the study sample identified their race or ethnicity as White or Caucasian (82.7%) and a smaller percentage as Black or African American (13.0%) compared to the recruitment sample. The racial breakdown of the recruitment sample more accurately reflects state-level estimates indicating 71.6% of North Carolina residents aged 18 and older identify as White or Caucasian and 21.9% identify as Black or African American [17].

### Data analysis

#### Analytic approaches

We generated descriptive statistics to investigate the prevalence of reporting transportation “difficulties” or “problems”, which we collectively refer to as “barriers,” that resulted in having arrived late to, delayed, or missed care. We quantified these barriers and health care outcomes based on respondents’ individual and household characteristics and reported the unadjusted association using Fisher’s exact test. We then conducted multivariate binomial logistic regressions to better understand the adjusted associations of individual, household, and geographic characteristics with transportation barriers that resulted in negative health care outcomes.

#### Independent variables

We collected information on individual and household-level variables known to influence travel behavior and people’s experiences using transportation and health care. Of particular interest to this study, we asked respondents to report how many times they went in person to medical appointments or treatments in the past year. Appointment frequency affects the likelihood of late arrivals and has been shown in previous studies to be associated with missed appointments [18]. We collected demographic information on respondents’ age, gender, race, and ethnicity. For statistical tests and regression analyses, we grouped respondents into two age bins: 18–64 years and 65 years or older. We also combined race/ethnicity categories to report race as White or Non-White; respondents who described themselves as “White” (regardless of whether they also identified as another race or as Hispanic, Latino, or Spanish) were included in White and those who did not describe themselves as “White” were included in Non-White. Respondents were asked to report whether they had a “disability or chronic condition that limits your daily activities”; those who replied “Yes” to this question were considered to have a disability in our analyses. We also asked respondents to identify what type(s) of health insurance they had.

Respondents were further asked to share their home ZIP code as well as to report how many motor vehicles are available for use by people in their household. In our analyses, household vehicles were classified as “None” for those who reported zero vehicles available for use in their household and “One or more” for those who reported at least one vehicle was available. We used data provided by CDW-H on the location of UNC Health clinics (including UNC Physicians Network doctor’s offices) to calculate the number of medical clinics in respondents’ home ZIP codes.

#### Outcome measures

We utilized four binary outcome measures to indicate how transportation barriers impacted healthcare usage and access. Respondents reported on whether transportation problems resulted in one of several health care outcomes of interest occurring in the past year: (1) delaying the scheduling of a medical appointment or treatment, (2) missing a medical appointment or treatment, (3) arriving more than 20 minutes late to a medical appointment or treatment, or (4) experiencing any of these three concerns. Delayed care and missed appointments have been linked with numerous negative consequences for patients, including increased hospitalizations, additional visits to emergency departments, and poorer long-term health outcomes [7, 9, 19]; late patient arrivals may have consequences such as disrupted clinic service operations and decreased overall service quality for patients [20].

#### Transportation barriers

Using data from questions asking respondents to elaborate on “transportation problems” that caused them to arrive late to, delay, or miss care, we identified commonly-reported transportation barriers. We further investigated and characterized these barriers by analyzing answers to open-ended text response questions. We used a thematic analysis approach [21] to code these responses in Dedoose, a web-based application for analyzing qualitative and mixed methods research with text data. The use of such qualitative techniques in travel behavior studies has been effective for adding depth and richness to findings on the subjective experiences of individuals related to using transportation [22].

## Results

### Prevalence of transportation barriers to health care

Among our study sample, 35.3% (*N* = 114) and 18.3% (*N* = 59) of respondents reported having delayed or missed medical appointments or treatments in the past year, respectively, because of transportation barriers; 16.4% (*N* = 53) of respondents reported having arrived more than 20 minutes late to a medical appointment or treatment in the past year because of transportation problems; and 39.0% (*N* = 126) experienced at least one of these outcomes (Table 2). Prevalence of transport barriers varied significantly with demographic, household, and spatial characteristics (Table 2). Individuals 65 and older, males, people without disabilities, and individuals with household vehicle access reported lower rates of transport barriers across the four measures. Individuals with no medical clinics in their home ZIP code were more likely to report being late and delaying or missing care. Individuals with higher numbers of medical appointments were also more likely to report being late due to transport barriers.

**Table 2:** Prevalence of having arrived late, delayed care, or missed care due to transport barriers by covariates (*N* = 323)

	Arrived Late	Delayed Care	Missed Care	Late, Delayed, or Missed Care
**All**	53 (16.4%)	114 (35.3%)	59 (18.3%)	126 (39.0%)
**Age**	***	***	***	***
18–64	38 (30.4%)	67 (53.6%)	45 (36.0%)	74 (59.2%)
65 plus	15 (7.6%)	47 (23.7%)	14 (7.1%)	52 (26.3%)
**Gender**		***		***
Female	36 (19.3%)	78 (41.7%)	39 (20.9%)	87 (46.5%)
Male	17 (12.5%)	36 (26.5%)	20 (14.7%)	39 (28.7%)
**Race**			*	
White	42 (15.7%)	91 (34.1%)	44 (16.5%)	103 (38.6%)
Non-White	11 (19.6%)	23 (41.1%)	15 (26.8%)	23 (41.1%)
**Disability**	***	***	***	***
Has one or more disabilities	48 (22.7%)	97 (46.0%)	51 (24.2%)	107 (50.7%)
Has no disability	5 (4.5%)	17 (15.2%)	8 (7.1%)	19 (17.0%)
**Household Vehicle(s)**	**	***	***	***
No household vehicle	7 (38.9%)	16 (88.9%)	10 (55.6%)	17 (94.4%)
Has household vehicle(s)	46 (15.1%)	98 (32.1%)	49 (16.1%)	109 (35.7%)
**Medical clinics in home ZIP**	**	*	***	**
0	26 (25.7%)	45 (44.6%)	32 (31.7%)	51 (50.5%)
1–5	17 (12.5%)	42 (30.9%)	14 (10.3%)	46 (33.8%)
6–10	8 (15.4%)	19 (36.5%)	9 (17.3%)	21 (40.4%)
11 plus	2 (5.88%)	8 (23.5%)	4 (11.8%)	8 (23.5%)
**Appointments in Past Year**	**			
1–5	12 (13.6%)	29 (33.0%)	15 (17.0%)	32 (36.4%)
6–10	13 (11.3%)	41 (35.7%)	16 (13.9%)	43 (37.4%)
11–15	9 (17.6%)	16 (31.4%)	11 (21.6%)	18 (35.3%)
16 plus	19 (27.5%)	28 (40.6%)	17 (24.6%)	33 (47.8%)

Note: * *p* < 0.1; **p < 0.05; *** *p* < 0.01 based on Fisher’s exact test

### Regression analysis of the relationship between demographics and transportation barriers

In a series of binomial regression analyses, we further tested the association between individual, household, and geographic characteristics and transportation barriers resulting in negative care outcomes. Looking at our adjusted models in Table 3, we found the likelihood of having arrived late to, delayed, or missed a medical appointment or treatment in the past year because of transportation barriers was significantly higher for younger adults aged 18–64 compared to older adults aged 65 and over. Not having a disability was associated with lower odds of having arrived late to or delayed care as well as the combined outcome. Having household vehicle(s) was similarly significantly associated with a reduced probability of having delayed or missed care. The number of medical clinics in the home ZIP, the number of appointments in the past year, gender, and race were not significantly associated with the probability of limiting care due to transport barriers.

**Table 3:** Binomial logit regression results (*N* = 323)

	Arrived Late	Delayed Care	Missed Care	Late, Delayed, or Missed Care
**Age** (ref: Aged 65 plus)				
Aged 18–64	3.30^***^ (1.55, 7.27)	1.98^**^ (1.09, 3.62)	4.97^***^ (2.32, 11.16)	2.19^**^ (1.21, 4.02)
**Gender** (ref: Female)				
Male	0.91 (0.44, 1.84)	0.65 (0.37, 1.12)	1.13 (0.56, 2.27)	0.58^*^ (0.33, 1.00)
**Race** (ref: White)				
Non-White	0.81 (0.34, 1.85)	0.68 (0.32, 1.38)	0.93 (0.40, 2.05)	0.49^*^ (0.23, 1.02)
**Disability** (ref: Has one or more disabilities)				
Has no disability	0.29^**^ (0.09, 0.74)	0.27^***^ (0.14, 0.52)	0.49 (0.19, 1.17)	0.26^***^ (0.13, 0.49)
**Household vehicle(s)** (ref: No household vehicle)				
Has household vehicle(s)	0.46 (0.15, 1.45)	0.07^***^ (0.01, 0.26)	0.24^**^ (0.08, 0.71)	0.03^***^ (0.002, 0.19)
**Medical clinics in home ZIP** (ref: 1–5)				
0	1.66 (0.79, 3.54)	1.25 (0.68, 2.31)	2.88^***^ (1.35, 6.38)	1.39 (0.75, 2.57)
6–10	1.61 (0.58, 4.28)	1.41 (0.66, 2.97)	2.41^*^ (0.86, 6.65)	1.53 (0.72, 3.23)
11 plus	0.60 (0.09, 2.47)	0.87 (0.32, 2.18)	1.94 (0.48, 6.64)	0.76 (0.28, 1.92)
**Appointments in past year** (ref: 6–10)				
1–5	1.26 (0.51, 3.13)	0.89 (0.46, 1.75)	1.26 (0.53, 3.00)	0.98 (0.50, 1.93)
11–15	2.05 (0.74, 5.54)	0.88 (0.40, 1.89)	2.14 (0.81, 5.61)	0.97 (0.45, 2.08)
16 plus	2.61^**^ (1.11, 6.29)	0.86 (0.43, 1.71)	1.80 (0.75, 4.33)	1.07 (0.53, 2.13)
Constant	0.15^**^ (0.03, 0.67)	9.45^**^ (2.00, 71.18)	0.16^**^ (0.03, 0.68)	20.72^***^ (3.23, 414.8)
Pseudo-R^2^ (McFadden)	0.17	0.16	0.20	0.19

Notes: * *p* < 0.1; **p < 0.05; *** *p* < 0.01. Odds ratios reported, followed by 95% confidence intervals in parentheses

### Explaining transportation barriers

15.2% (*N* = 49) of respondents reported that the cost of traveling prevented them from going to a medical appointment, while 20.7% (*N* = 67) reported that not having a ride posed a barrier to seeking or reaching care. When asked to elaborate on which costs contributed to transportation barriers, respondents mentioned the costs of gasoline; parking; fares for public transportation, taxis, and app-based ridehailing services like Uber and Lyft; paying a friend to drive them or reimbursing someone for gasoline or car use; tolls; and buying meals and lodging while traveling for care. One respondent wrote, considering the cost calculus of getting to a medical appointment at the hospital, “It costs 3/4 tank of fuel, $28, to do a round trip to the hospital plus $12 for parking. If I don’t schedule my appointments the right way, sometimes money is very tight when my monthly check is running out before I get the next one.” Another respondent similarly explained that transportation costs could become prohibitive considering other finances, writing, “Taxi fares are expensive. ... I’m on a fixed income and don’t have but 100 [dollars] left after rent and utilities to pay for [my] medication copay and transportation.”

Respondents who reported not having a ride posed a barrier to getting care explained that they did not have a ride for a number of reasons related to driver availability, or not having access to someone who could drive them at the time of their appointments or treatments; car availability, contingent on whether a car they had access to was working or whether they typically had access to a vehicle at all; the availability of alternative transportation services, including public or community transportation; and scheduling issues associated with using demand response transportation services (e.g., dial-a-ride, paratransit). Respondents also mentioned that traffic, construction or unexpected delays, and inclement weather contributed to not having a ride to a medical appointment because their driver did not show up or to arriving late to scheduled appointments.

Often, respondents reported that a combination of barriers kept them from reaching care—some related to transportation costs and driver or car availability and others related to their state of health. In this way barriers were complicated by conditional access to transportation as well as changes in people’s ability to travel for care. One respondent explained they could not get to a recent appointment because, “We have one vehicle. My partner could not get off from work to take me [to the appointment]. If a car had been available, I do not feel very comfortable driving myself with the medications I take.” Another recounted a recent trip to a doctor’s appointment in which weather, driver availability, and their health all contributed to difficulties: “It started raining and I could not find a driver. I tried to drive but had a vertigo spell so ended up pulling over.” Fortunately, they wrote, after pulling over, “I called the office. My provider was able to talk to me on the phone and my husband rescued me later in the day.” While this instance may not have substantially interrupted this respondent’s care, it highlights the complex, compounding, circumstantial difficulties that can contribute to transportation barriers.

## Discussion

Approximately 1 in 3 respondents in our study sample reported having experienced transportation barriers between June 2020 and June 2021 that resulted in having arrived late to, delayed, or missed a medical appointment or treatment. This is notably higher than previous studies have found for similar health care user populations. Wolfe et al. found that of US adults aged 19 plus who self-reported having a “poor” health status and who had made 4 or more emergency department visits in the past year, 11.6% and 11.9% had delayed care due to lack of transportation [2]. We expect that our sample of high-frequency health care users has both greater health care needs and health care-related transportation burdens, likely exacerbated by the COVID-19 pandemic. It is problematic, then, that transportation barriers affected a substantial number of respondents. Costs of traveling for medical appointments and a lack of driver or car availability emerged in our study as major transportation barriers to health care. However, respondents explained that transportation barriers were often complex, involving circumstantial problems related to one’s ability to access and pay for transportation as well as their personal health, which, in some cases, compromised people’s ability to travel in a particular way (i.e., as a driver) or entirely.

Transportation barriers were experienced unequally. Results indicate that younger adults aged 18–64, people without vehicle access, and people with disabilities were significantly more likely to encounter transportation barriers resulting in having arrived late to, delayed, or missed medical appointments or treatments regardless of how many appointments they had.

All respondents in our sample were enrolled in Medicaid or Medicare. While most respondents were aged 65 and older and had Medicare (*N* = 204), a subset were adults aged 18–64 enrolled in Medicaid (*N* = 73), Medicare (*N* = 92), or dual-enrolled in both public insurance programs (*N* = 35). Consistent with previous research, we found these respondents—younger adults enrolled in Medicaid or Medicare—were more likely to encounter transportation barriers to health care [2, 5]; this may be because they have low incomes and may experience other socioeconomic and transportation disadvantages. As high-frequency health care users enrolled in public health insurance programs, these respondents also likely experience more health-related disadvantages—for instance, they may have disabilities or chronic conditions, or may generally be in poorer health—that keep them from accessing reliable transportation and consistent care. In our study, of the 135 respondents aged 18–64, 88.1% (*N* = 119) had a disability.

These findings are interesting given Medicaid members and adults with disabilities enrolled in Medicare should qualify for the NEMT benefit and be eligible to use paratransit, respectively; both NEMT and paratransit services are intended to reduce transportation barriers. Our findings suggest high-frequency health care users aged 18–64, who were more likely to report encountering transportation barriers, may not be aware of these services or otherwise may not use them. It is possible that the circumstantial, potentially transient nature of transportation barriers may contribute to more barriers resulting in negative health care outcomes. For example, individuals who sometimes have access to a household vehicle or driver may not think to seek alternatives or plan back-up transportation for when they do not have a car or driver available. Similarly, an individual who can usually drive themselves to medical appointments or treatments may not be aware of alternative arrangements for when they cannot due to illness or injury. Even if they are aware of alternatives, such as NEMT or transit/paratransit offerings, these services must typically be scheduled in advance.

Our findings must be considered in the context of the COVID-19 pandemic, which exacerbated transportation barriers, particularly those resulting in delayed care, in part by reducing transportation and health care availability. Chen et al. detailed ways in which the pandemic has affected transportation access to health care and found that people generally needed extra help with trips to care; furthermore, people with elevated health risks as well as low-income individuals and people of color have been disproportionately burdened by transportation barriers [10]. Our findings support these conclusions and provide more evidence that the pandemic is likely exacerbating transportation and health disparities that disadvantage people that may need to seek care more, such as those with disabilities and chronic conditions, as well as those who use public insurance programs.

Though our study sample is not statistically representative, findings from this research shed light on transportation barriers that may be generalizable, particularly to other high-frequency health care users enrolled in Medicaid or Medicare. As we found that younger adults enrolled in public health insurance programs and individuals with disabilities in our sample were more likely to encounter transportation barriers, it is likely that the findings of our study underestimate the prevalence of transportation barriers; people with low incomes, disabilities, and those in very poor health are often underrepresented in survey studies and may not have access to the technology required to complete a web-based questionnaire [23–25]. Furthermore, rural residents are known to encounter more transportation barriers and have less internet access [26], and were underrepresented in our study sample; only 12.4% of respondents (*N* = 40) lived in non-metropolitan areas. More investigation is needed of transportation barriers affecting individuals likely to experience compounding transportation and health disadvantages, including individuals enrolled in Medicaid, younger adults with disabilities enrolled in Medicare, and people living in rural areas.

## Conclusions and recommendations

We offer recommendations that might address the complex transportation barriers that affect high-frequency health care users and disproportionately burden younger adults aged 18–64 enrolled in public health insurance programs. First, echoing Chen et al., we recommend more coordination between transportation and health professionals and the implementation of programs to expand and improve patient awareness of medical transportation programs, including the NEMT benefit, paratransit services, and others [10]. Ensuring patients have the information they need to access care is particularly important during this time of health crisis. Communicating health and medical transportation information to those who need it should be prioritized at points of care (e.g., doctor’s offices, hospital-based outpatient clinics, dialysis centers, etc.) and through established transportation and medical communications channels such as transportation reservation lines and patient listservs [14]. Medical providers could also make this information available during telehealth appointments and using patient engagement platforms like patient portals and mobile applications, which patients may have become more familiar with during the pandemic.

Second, we recommend that transportation and health entities address major transportation barriers, including transportation costs and availability. This might be done by providing subsidies for expenses such as gasoline and parking, which respondents in our study indicated could be prohibitive to seeking health care. These would likely be best coordinated between transportation and medical stakeholders, including health insurance plans, medical providers, and transportation providers. To improve medical transportation availability, we recommend that transportation and health care entities explore adopting emerging technologies and participating in innovative collaborations to provide or expand health care mobility services. Wolfe and McDonald identified three popular approaches for this, including health care providers leveraging app-based ridehailing technology to book patient trips; health plans partnering with ridehailing companies to expand transportation offerings to beneficiaries; and transit/paratransit providers partnering with ridehailing companies to offer more flexible services [27]. Resources might also be directed to improve existing transit services or planned public transportation projects to facilitate access to medical clinics [28]. Health care providers should also consider solutions that link disadvantaged households to health services by “decentralizing care,” or building up health service infrastructure in local institutions so that they can serve people in surrounding neighborhoods [29]. Creating satellite or mobile clinics that can run out of local pharmacies, housing complexes, and schools, for example, is a way to disperse health resources, reduce transportation burdens associated with seeking care, and generally expand access to care, particularly in underserved communities.

Third, we recommend that transportation and health entities direct resources to address transportation barriers equitably, as our findings concord with those of other studies showing transportation barriers and negative health care outcomes are not experienced evenly. Our results suggest that more attention should be given to alleviating transportation barriers among adults aged 18–64 enrolled in public health insurance programs and individuals with disabilities. Members of these groups may already qualify for targeted transportation assistance programs such as NEMT and paratransit, but they may not be aware of them. These and other transportation programs also may not be accessible to those who need them; for instance, people with certain disabilities may require wheelchair-accessible vehicle services and individuals without access to internet-enabled devices may need to schedule transportation by phone. Policies and programs to address transportation barriers to care must be designed with accessibility and equity as guiding tenets to serve individuals seeking care most effectively and ultimately promote transportation and health care access.

## Data Availability

The datasets generated and/or analysed during the current study are not publicly available to protect respondents’ privacy; de-identified data may be available from the corresponding author on reasonable request.

## Abbreviations

CDW-H: Carolina Data Warehouse for Health
NEMT: Non-emergency medical transportation

## References

[1] Syed ST, Gerber BS, Sharp LK (2013). Traveling towards disease: transportation barriers to health care access. J Community Health.

[2] Wolfe MK, McDonald NC, Holmes GM (2020). Transportation barriers to health Care in the United States: findings from the National Health Interview Survey, 1997–2017. Am J Public Health.

[3] Silver D, Blustein J, Weitzman BC (2012). Transportation to clinic: findings from a pilot clinic-based survey of low-income suburbanites. J Immigr Minor Health.

[4] Ryvicker M, Bollens-Lund E, Ornstein KA (2020). Driving status and transportation disadvantage among Medicare beneficiaries. J Appl Gerontol.

[5] Cheung PT, Wiler JL, Lowe RA, Ginde AA (2012). National Study of barriers to timely primary care and emergency department utilization among Medicaid beneficiaries. Ann Emerg Med.

[6] Hwang AS, Atlas SJ, Cronin P, Ashburner JM, Shah SJ, He W (2015). Appointment “no-shows” are an independent predictor of subsequent quality of care and resource utilization outcomes. J Gen Intern Med.

[7] Kangovi S, Barg FK, Carter T, Long JA, Shannon R, Grande D (2013). Understanding why patients of low socioeconomic status prefer hospitals over ambulatory care. Health Aff (Millwood).

[8] Kheirkhah P, Feng Q, Travis LM, Tavakoli-Tabasi S, Sharafkhaneh A (2016). Prevalence, predictors and economic consequences of no-shows. BMC Health Serv Res.

[9] Nguyen DL, DeJesus RS (2010). Increased frequency of no-shows in residents’ primary care clinic is associated with more visits to the emergency department. J Prim Care Community Health.

[10] Chen KL, Brozen M, Rollman JE, Ward T, Norris KC, Gregory KD, et al. How is the COVID-19 pandemic shaping transportation access to health care? Transp Res Interdisc Perspect. 2021;10:100338. 10.1016/j.trip.2021.100338 PMC8422279 34514368

[11] Giannouchos TV, Brooks JM, Andreyeva E, Ukert B (2022). Frequency and factors associated with foregone and delayed medical care due to COVID-19 among nonelderly US adults from august to December 2020. J Eval Clin Pract.

[12] Byrnes ME, Brown CS, De Roo AC, Corriere MA, Romano MA, Fukuhara S (2021). Elective surgical delays due to COVID-19. Med Care.

[13] Parker MEG, Li M, Bouzaghrane MA, Obeid H, Hayes D, Frick KT (2021). Public transit use in the United States in the era of COVID-19: transit riders’ travel behavior in the COVID-19 impact and recovery period. Transp Policy.

[14] Cochran AL (2020). Impacts of COVID-19 on access to transportation for people with disabilities. Transp Res Interdiscip Perspect..

[15] Wang J, McDonald N, Cochran AL, Oluyede L, Wolfe M, Prunkl L (2021). Health care visits during the COVID-19 pandemic: a spatial and temporal analysis of mobile device data. Health Place.

[16] U.S. Census Bureau (2019). Age and sex, 2015–2019 American community survey 5-year estimates.

[17] U.S. Census Bureau (2019). Citizen, voting-age population by selected characteristics, 2019 American community survey 1-year estimates.

[18] Cashman SB, Savageau JA, Lemay CA, Ferguson W (2004). Patient health status and appointment keeping in an Urban Community health center. J Health Care Poor Underserved.

[19] Weissman JS, Stern R, Fielding SL, Epstein AM (1991). Delayed access to health care: risk factors, reasons, and consequences. Ann Intern Med.

[20] Glowacka KJ, May JH, Goffman RM, May EK, Milicevic AS, Rodriguez KL (2017). On prioritizing on-time arrivals in an outpatient clinic. IISE Trans Healthc Syst Eng.

[21] Braun V, Clarke V (2006). Using thematic analysis in psychology. Qual Res Psychol.

[22] Mars L, Arroyo R, Ruiz T (2016). Qualitative research in travel behavior studies. Transp Res Procedia.

[23] Perrin R (2021). Atske S.

[24] Roberts BW, Yao J, Trzeciak CJ, Bezich LS, Mazzarelli A, Trzeciak S (2020). Income disparities and nonresponse Bias in surveys of patient experience. J Gen Intern Med.

[25] Cheung KL, Ten Klooster PM, Smit C, de Vries H, Pieterse ME (2017). The impact of non-response bias due to sampling in public health studies: a comparison of voluntary versus mandatory recruitment in a Dutch national survey on adolescent health. BMC Public Health.

[26] Vogels E. a. some digital divides persist between rural, urban and suburban America. Pew Res Center. https://www.pewresearch.org/fact-tank/2021/08/19/some-digital-divides-persist-between-rural-urban-and-suburban-america/. Accessed 16 Dec 2021.

[27] Wolfe MK, McDonald NC (2020). Innovative health care mobility services in the US. BMC Public Health.

[28] Smith LB, Yang Z, Golberstein E, Huckfeldt P, Mehrotra A, Neprash HT (2022). The effect of a public transportation expansion on no-show appointments. Health Serv Res.

[29] Butler SM (2020). Four COVID-19 lessons for achieving health equity. JAMA.

